# Toward universal dose prediction: A multi‐scale, multi‐objective framework for sequential boost radiotherapy

**DOI:** 10.1002/mp.70523

**Published:** 2026-06-08

**Authors:** Austen Matthew Maniscalco, Xinran Zhong, Sean Domal, Yu‐Chen Lin, David Sher, Mu‐Han Lin, Steve Jiang, Dan Nguyen

**Affiliations:** ^1^ Medical Artificial Intelligence and Automation Laboratory Department of Radiation Oncology University of Texas Southwestern Medical Center Dallas Texas USA

**Keywords:** artificial intelligence, boost, deep learning, dose prediction, Jacobian descent, multi‐plan, multi‐scale, multi‐task, radiation therapy, radiotherapy, sequential, universal

## Abstract

**Background:**

Sequential boost radiotherapy (RT) poses a challenge in allocating dose across multiple plans while protecting organs at risk (OARs). Clinicians must decide whether OAR sparing should occur primarily in the initial plan, the boost plan(s), or all plans, resulting in a time‐intensive, iterative optimization process.

**Purpose:**

Current dose prediction frameworks are limited to single plans and do not account for complexities introduced by sequential boosts. We propose a multi‐plan dose prediction framework that models both individual plan doses and the cumulative plan‐sum dose. By integrating the full course's context, this approach may help planners establish optimization objectives with fewer iterative adjustments. Ultimately, this framework aims to enable a versatile dose prediction approach adaptable to any RT course, regardless of treatment site, fractionation scheme, or the number of sequential plans.

**Methods:**

We developed a U‐Net‐based Hybrid Convolutional Neural Network (CNN) that processes CT images, OARs, PTVs, and dosimetric goals to predict dose distributions for each plan and the plan‐sum. It incorporates five pooling layers, skip connections, and a transformer bottleneck to capture global context. Deep supervision is applied in the decoder to encourage robust feature representation at deeper network layers, acting as a form of regularization. The single‐plan model used for comparison differs from the multi‐plan model in several key ways: (1) it only processes a single treatment plan at a time, (2) plan‐sum dosimetric goals are re‐scaled to reflect the plan fractions, (3) plan‐sum PTVs are omitted, (4) it only predicts plan doses, and (5) plan‐sum dose distributions were generated by summing plan dose predictions. Models were trained using a multi‐objective loss vector of Mean Squared Error (MSE) for voxel‐wise accuracy and Multi‐Scale Structural Similarity Index (MS‐SSIM) for regional coherence. Loss was averaged across all output levels to encourage global coherence. To improve training stability and ensure that parameter updates benefit both loss objectives, we used a Jacobian descent strategy via the TorchJD package. We collected a site‐agnostic dataset of 64 patients that underwent sequential boost RT (38/6/20 training/validation/testing split).

**Results:**

The multi‐plan model and single‐plan model were trained to convergence. Evaluation metrics included Mean Absolute Error as a percent of prescription dose (MAE/Rx) and Structural Similarity Index Measure (SSIM). The multi‐plan model achieved statistically significant differences versus the single‐plan model in plan dose distributions with MAE/Rx (1.244 ± 0.151% vs. 1.650 ± 0.172%, *p *< 0.001) and SSIM (0.964 ± 0.005 vs. 0.944 ± 0.009, *p* < 0.001), and plan‐sum dose distributions with MAE/Rx (1.146 ± 0.174% vs. 1.525 ± 0.188%, *p *< 0.001) and SSIM (0.972 ± 0.006 vs. 0.960 ± 0.006, *p* < 0.001).

**Conclusion:**

Our multi‐plan dose prediction framework improves voxel‐wise accuracy and perceptual consistency by incorporating plan‐sum information. Unlike traditional single‐plan prediction models, our approach processes data from multiple treatment plans simultaneously, allowing it to consider cumulative dose requirements across the full treatment course. This approach can streamline treatment planning by providing clinicians with an accurate, comprehensive strategy for dose allocation in sequential boost RT. This framework lays the foundation for a universal RT dose prediction model capable of handling any fractionation scheme, disease site, or number of plans, which we plan to demonstrate in future work.

## 1 INTRODUCTION

Radiation therapy (RT) treatment planning remains a time‐intensive, iterative process, requiring careful navigation of trade‐offs between planning target volume (PTV) coverage and organ‐at‐risk (OAR) sparing. These challenges are exacerbated in sequential boost radiotherapy, where treatment is delivered across multiple stages (e.g., initial and boost plans) and cumulative dose constraints must be respected across the entire treatment course. This interdependence of plans often necessitates repeated cycles of individual plan optimization followed by plan‐sum dose evaluation, until all individual plan doses and the plan‐sum dose satisfy clinical directives. For example, when OAR sparing conflicts with PTV coverage, it may be particularly difficult to determine whether PTV under‐coverage should be concentrated in a single plan or evenly distributed across all plans. This highlights the need for a tool that can pre‐emptively guide planners in the optimal allocation of dose across all plans in a patient's course of treatment.

Knowledge‐based planning (KBP) emerged as a method to leverage historical plan data by constructing predictive models that estimate achievable dose‐volume histogram (DVH) curves for new patients based on anatomical similarities to previously treated cases.^1^ The approach relies on extraction of anatomical features from a cohort of carefully selected cases, including dosimetric and geometric features for each OAR, to fit a regression model that can estimate cumulative DVHs for OARs in new patients.^2^, ^3^ However, these features represent oversimplifications of complex three‐dimensional anatomical relationships. They focus predominantly on pairwise interactions between individual organs and the target while neglecting the multidimensional spatial context that dose distributions calculations natively consider. Furthermore, KBP systems predict only DVH curves rather than full three‐dimensional dose distributions, providing an incomplete description of plan quality. Even when DVH predictions are accurate, achieving such distributions through inverse optimization is not guaranteed and may converge to inferior plans as compared to clinical baseline plans.^4^

Deep learning‐based models followed shortly after, in which dose predictions may be used to guide and accelerate the treatment planning process. Song et al. introduced an information‐aided planning scheme in which predicted dose values populated initial optimization objectives, reducing manual optimization time by nearly 50% and improving certain dosimetric metrics compared to the control group.^5^ Similarly, Gronberg et al. demonstrated that deep learning‐based dose prediction can support automated quality assurance by enabling planners to conduct comparative reviews to improve the quality and consistency of head and neck (H&N) treatment plans.^6^

Despite advances in neural network architecture design, most existing dose prediction models remain limited in clinical application because they are tailored to specific disease sites or protocols. Zhang et al. proposed a conditional generative adversarial network (cGAN)‐based model, but it was trained exclusively on cervical cancer patients receiving 54 Gy in 30 fractions with three coplanar arcs.^7^ Hu et al. developed a transformer‐based ensemble for dose prediction, but restricted their dataset to Head and Neck patients receiving 70 Gy in 35 fractions with a limited set of seven OARs.^8^ Fu et al. introduced a diffusion model for dose prediction and benchmarked it against several state‐of‐the‐art (SOTA) methods, but their evaluation was limited to thoracic tumor patients.^9^

More recent studies have begun addressing variability in prescription schemes. Duan et al. evaluated the performance of their dose prediction model on a cohort of esophageal patients with some variation in dose and fractionation (e.g., 41.4 Gy/23 fx, 50.4 Gy/28 fx, 50.4 and 60.2 Gy/28 fx).^10^ Cao et al. demonstrated feasibility of in predicting dose in simultaneous integrated boost (SIB) cases with varying fractionation schemes and dose levels.^11^ Proton dose prediction has also been investigated, with distinct challenges in range uncertainty and linear energy transfer modeling. Vasquez et al. demonstrated proton dose prediction across multiple anatomical sites, though their approach wasn't focused on generalization as it relied on separately trained models for each disease site.^12^

Beyond direct prediction approaches, reinforcement learning (RL) agents offer an alternative route toward AI‐guided treatment planning by iteratively adjusting optimization objectives to reproduce target dose distributions based on explicit DVH goals. Clinical adoption of RL agents remains limited by the difficulty of designing generalizable rewards, scaling the action space across diverse objective update possibilities, and ensuring interpretability of learned actions as described by Shen et al. ^13^ Whereas RL agents act on a state space of DVH‐derived metrics, dose prediction models operate on full spatial information, and their predictions serve as an achievable benchmark for RL reward computation. Agent actions could be scored against a concrete target rather than against iterative scalar‐reward optimization that lacks clear termination criterion. Dose prediction and RL thus present a potential synergy for fully automated treatment planning, with dose prediction supplying a spatial goal that RL iteratively optimizes towards.

In pursuit of broader dose prediction model generalization, our previous work introduced a multi‐task architecture capable of predicting dose across multiple modalities simultaneously, leveraging a strategy that is both prescription‐agnostic and site‐agnostic.^14^ To our knowledge, no prior dose prediction work has demonstrated generalization across treatment sites within a single model. Conventional approaches require fixed, site‐specific OAR inputs (e.g., parotids for head‐and‐neck, lungs for thoracic cases), inherently limiting applicability to the anatomical sites on which they were trained. Our approach addresses this by encoding planning objectives into a goal array, where each voxel is assigned a dose value based on its associated OAR sparing constraint rather than its anatomical identity. This decouples the model from site‐specific anatomy and may enable generalization to any treatment site where planning objectives can be defined. In this work, we extend the goal array to three channels representing maximum, mean, and volumetric dosimetric objectives, ensuring that all constraint types are represented without requiring consolidation into a single channel.

Despite these advances in site‐agnostic prediction, existing models remain limited to single‐plan prediction and do not address the complexities introduced by multi‐plan treatment courses, such as those involving sequential boosts. In these scenarios, distribution of dose must be coordinated across multiple plans that may each have different PTVs, fractionation schemes, and clinical priorities. Furthermore, the extent of OAR sparing often varies between treatment phases, making it difficult for planners to anticipate the optimal distribution of dose across plans to achieve a clinically acceptable plan‐sum. This typically necessitates a time‐consuming, iterative process of plan‐by‐plan optimization and plan‐sum dose evaluation to achieve the desired balance between PTV coverage and OAR sparing.

To address these challenges, we propose a multi‐plan dose prediction framework for sequential boost radiotherapy that jointly models individual plan doses and the cumulative plan‐sum dose. We evaluate this approach on a multi‐site dataset comprising head and neck (*n* = 37), prostate (*n* = 11), pelvis (*n* = 8), bladder (*n* = 3), bony metastases (*n* = 3), and lung (*n* = 2) cases treated with sequential boost radiotherapy.

The architecture integrates a multi‐scale design with deep supervision and employs a Jacobian descent strategy for gradient aggregation, enabling stable and synergistic multi‐objective learning of both local and global features. This design drives accurate, context‐aware modeling of dose across the entire treatment course, allowing planners to make informed, data‐driven decisions at the outset of the planning process. By capturing inter‐plan dose trade‐offs and spatial dependencies, the multi‐plan model may reduce dose prediction error as compared to a model that lacks—plan‐sum information in its input. In addition, a multi‐plan model can support a more efficient optimization workflow for sequential boost RT that reduces reliance on trial‐and‐error adjustments. More broadly, this work establishes the foundation for a universal, generalizable dose prediction model that can handle any number of plans, fractionation schemes, or disease sites within a unified framework.

## 2 METHODS

### 2.1 Network architecture

**FIGURE 1:**
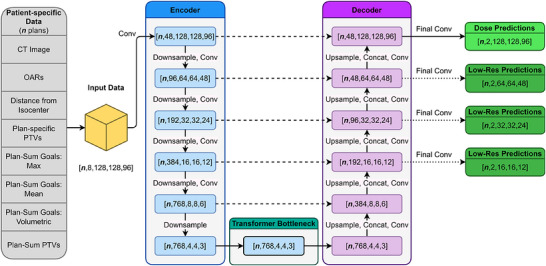
Overview of the proposed multi‐plan dose prediction network architecture. The model processes input data for *n* plans, each represented by eight channels: CT image, OARs, distance‐from‐isocenter map, plan‐specific PTVs, three plan‐sum goal channels (maximum, mean, and volumetric), and plan‐sum PTVs. The encoder consists of five levels of sequential convolution and downsampling, followed by a transformer bottleneck to capture global spatial context. The decoder mirrors the encoder with five levels of upsampling and sequential convolution, including skip connections (dashed arrows) from the encoder. Deep supervision paths (dotted lines) generate predictions at multiple resolutions, and are only employed during model training. The final output contains two channels: the first corresponds to the predicted plan dose, and the second to the predicted plan‐sum dose.

We designed a multi‐plan dose prediction network based on a hybrid 3D U‐Net architecture with a transformer bottleneck, as illustrated in Figure 1, and implemented it using the PyTorch deep learning framework in Python. The U‐Net is a widely used encoder‐decoder architecture that progressively compresses spatial information and then reconstructs it, with skip connections that preserve fine‐grained details. Our hybrid design incorporates a transformer at the network's bottleneck, which has been suggested to improve the network's ability to capture global features as compared to a conventional U‐Net. Similar architectures demonstrated strong performance in ablation studies by Hu et al. and Xu et al. ^15^, ^16^

The model is designed to jointly predict dose distributions for individual plans and the cumulative plan‐sum. Input data is organized into eight channels: CT image, OARs, distance‐from‐isocenter map, plan‐specific PTVs, three plan‐sum goal channels (maximum, mean, and volumetric), and the plan‐sum PTVs. In goal channel generation, unspecified portions of OARs (regions without a dose sparing goal) were assigned to a constant value of 95% of the plan's prescription dose. For a given patient, the effective batch size *n* corresponds to the number of individual plans, enabling simultaneous processing of all plans in the treatment course. The model outputs two channels corresponding to predicted dose distributions for the plan and the full course (plan‐sum).

#### 2.1.1 Layer components

The network is composed of several reusable building blocks, described below. Readers less familiar with deep learning terminology may refer to Figure 1 for a visual overview of how these components connect.

**Sequential Convolutional Layers** perform two consecutive sequences of: 3D convolution (3 × 3 × 3 kernel), CCReLU activation, group normalization (16 groups of channels), and 3D DropBlock. CCReLU is a variant of the standard ReLU activation that concatenates the positive and negative components of the input, then applies convolution for channel reduction. This construction retains both activation phases and lets the 1 × 1 × 1 reduction convolution learn a sign‐aware channel embedding that a plain ReLU at matched width cannot represent, though this comes at the cost of a modest increase in parameters per block. DropBlock is a regularization technique that randomly zeroes contiguous regions during training to prevent overfitting. The DropBlock rate increases with network depth, up to a maximum of 0.1, and is defined as $0.1\times \surd \frac{level}{N}$, where level is the current depth and N is the total number of levels (here, *N* = 5).

**Downsampling Layers** reduce spatial resolution by a factor of 2× to compress information. Each layer performs three parallel operations using 2 × 2 × 2 kernels—3D max pooling, 3D average pooling, and 3D strided convolution (2 × 2 × 2 stride). These outputs are concatenated along the channel dimension—tripling the number of features—and restored to the original channel depth by passing through a sequence of 3D convolution (1 × 1 × 1 kernel), CCReLU, group normalization and DropBlock.

**Upsampling Layers** increase spatial resolution by a factor of 2× to reconstruct the dose. Each layer uses three parallel operations: nearest neighbor interpolation, trilinear interpolation, and 3D transposed convolution (2 × 2 × 2 kernel, 2 × 2 × 2 stride). It concatenates and compresses features identically to approach in Downsampling Layers.

The **Transformer Bottleneck** is composed of six transformer encoder layers, which specializes in modeling long‐range dependencies by allowing information from each spatial location to learn information from all other locations. The transformer encoder layer follows the PyTorch implementation of the approach by Vaswani et al. that uses self‐attention and a feedforward network, with the following configuration: 768 input features, 16 heads for multi‐head attention, a dropout rate of 0.1, GELU activation, and a feedforward dimension of 2048.^17^

**Final Convolutional Layers** use a 3D convolution (1 × 1 × 1 kernel) to reduce the number of feature channels to 2, followed by a linear activation function. This prodocues the final dose predictions: channel 1 for the individual plan dose, and channel 2 for the plan‐sum dose.

#### 2.1.2 Encoder

The encoder progressively compresses the input data across five levels, each composed of a Sequential Convolutional Layer followed by a Downsampling Layer. Skip connections are established between each encoder level and the corresponding decoder level, which allow the network to preserve high‐resolution features for later reconstruction.

Data flow proceeds as follows: The input tensor of shape [*n*, 8, 128, 128, 96] is passed through the first Sequential Convolutional layer, producing an output of [*n*, 48, 128, 128, 96], which is then downsampled to [*n*, 48, 64, 64, 48]. The second Sequential Convolutional layer produces an output of [*n*, 96, 64, 64, 48], and this process repeats until the final downsampled size of [*n*, 768, 4, 4, 3].

At the bottleneck, the encoded tensor [*n*, 768, 4, 4, 3] is flattened in its spatial dimensions and reshaped to [*n*, 4 × 4 × 3, 768]. Positional embeddings are added to preserve spatial context, and the resulting tensor is passed through the Transformer Bottleneck. The transformer encoder layers apply multi‐head self‐attention and feedforward operations to improve modeling of long‐range spatial dependencies. The output of the transformer, also of shape [*n*, 48, 768], is reshaped back to [*n*, 768, 4, 4, 3] and forwarded to the decoder.

#### 2.1.3 Decoder

The decoder reconstructs spatial resolution across five levels using Upsampling Layers, skip connections from the encoder, and Sequential Convolutional Layers. At each level, upsampled feature maps are concatenated with corresponding encoder features and refined through a Sequential Convolutional layer to reduce the channel dimension.

Decoding begins with the Transformer Bottleneck output of shape [*n*, 768, 4, 4, 3], which is upsampled to [*n*, 768, 8, 8, 6]. After concatenation with skip connection features, the combined tensor of shape [*n*, 1536, 8, 8, 6] is compressed to [*n*, 384, 8, 8, 6] via a Sequential Convolutional Layer. This process is repeated to progressively increase spatial resolution and reduce feature depth until the final decoder level produces [*n*, 48, 128, 128, 96]. This output is passed through a Final Convolutional Layer to generate the final dose predictions of shape [*n*, 2, 128, 128, 96].

During training, deep supervision is applied yielding outputs at the four shallowest decoder layers. Deep supervision is a training technique that has been shown to reduce time to convergence, stabilize training, and act as a form of regularization, such as in works by Ren et al. and Wang et al.^18^, ^19^ However, a common issue encountered deep supervision is that it can be challenging to predict a full resolution label from intermediate features. Therefore, our network modified each level to use a dedicated Final Convolutional Layer that outputs predictions at lower resolutions of 16 × 16 × 12, 32 × 32 × 24, and 64 × 64 × 48, followed by the final output of 128 × 128 × 96. Ground truth doses are downsampled to match each resolution and losses are computed independently for each level. Losses are then averaged to promote stable, multi‐scale learning of both global and local dose features. During inference, only the final output [*n*, 2, 128, 128, 96] is retained as the model's dose prediction.

#### 2.1.4 Single‐plan (Baseline) model modifications

**FIGURE 2:**
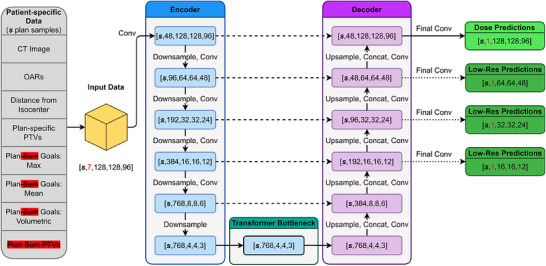
Overview of the single‐plan dose prediction network architecture, which serves as this study's baseline for comparison to the multi‐plan model. The model processes the same batch size as the proposed multi‐plan model, but draws *s* augmented samples from a randomly selected plan per patient during training. Input data consists of seven channels: CT image, OARs, distance‐from‐isocenter map, plan‐specific PTVs, and re‐scaled plan goals derived from the plan‐sum maximum, mean, and volumetric objectives. Plan‐sum PTVs are excluded. The architecture is nearly identical to the proposed multi‐plan model but is restricted to a single output channel corresponding to the predicted plan dose.

To evaluate the effectiveness of the proposed multi‐plan architecture, we implemented a single‐plan (baseline) model shown in Figure 2, with several key differences that restrict it to single‐plan processing.

First, during training, a single plan is randomly selected from each patient, and s augmented samples are drawn from that plan. The value of s is set equal to *n*, ensuring that both models are trained with the same effective batch size. Importantly, both models are trained on identical data; only the sampling strategy differs such that the single‐plan model performs parameter updates using the context of one plan at a time.

Second, the plan‐sum dosimetric goals (maximum, mean, and volumetric) are re‐scaled to reflect the fractionation scheme of the selected plans. For example, if the plan‐sum objectives correspond to 35 fractions and the selected plan is for 15 fractions, the goal values are scaled by a factor of 15/35.

Third, plan‐sum PTVs are omitted entirely from the input.

Lastly, the output at all decoder levels consists of a single channel corresponding to the predicted dose distribution for the selected plan. For evaluation purposes, the cumulative plan‐sum dose is not directly predicted by the network but is instead generated by summing the individually predicted doses for each plan after inference.

### 2.2 Dataset and preprocessing

A total of 64 patients with 131 clinically approved treatment plans were retrospectively included in this study. The dataset comprises all available patients at the time of this study that were specifically treated with sequential boost radiotherapy on our institution's O‐ring linear accelerator, a recently commissioned platform with dose distributions that best represent our most current planning practices. All treatment plans in this study used static IMRT 6MV‐FFF beams. To maximize dataset size and model generalizability, no restrictions were placed on disease site, number of fractions, prescription dose, or patient orientation. In total, there were 37 H&N, 2 lung, 8 pelvis, 3 bony metastases, 11 prostate, and 3 bladder cases. The dataset was randomly partitioned into training, validation, and testing subsets, as shown in Table 1.

**TABLE 1:** Dataset composition by cohort. A total of 131 plans across 64 patients were included, all of whom underwent sequential boost radiotherapy on an O‐ring linear accelerator.

Subset	Patients	Plans	Head and Neck (H&N)	Lung	Pelvis	Bony metastases	Prostate	Bladder
Training	38	77	21	1	5	2	7	2
Validation	6	13	4	0	1	0	1	0
Testing	20	41	12	1	2	1	3	1

While the dataset size is modest compared to single‐site studies, the site‐agnostic design benefits from this diversity. As described previously, the goal array formulation encodes planning objectives in place of fixed OAR‐specific input channels, which may enable knowledge learned from well‐represented sites to inform predictions for under‐represented sites.

All patients were treated with sequential boosts—however, a subset of plans also used the simultaneous boost (SIB) technique. For example, a H&N treatment course may deliver a SIB of 36 and 40 Gy in 20 fractions for the initial plan, and 30 Gy in 15 fractions for the boost plan. For these cases, the term “prescription dose” refers to the highest prescription dose delivered within the plan. Plan‐sum dosimetric goals were specified by institutional guidelines but were overridden by physician directives when available. Ground truth structures—including all GTV(s), CTV(s), PTV(s), and OAR(s)—and dose distributions were approved by board‐certified radiation oncologists.

All DICOM data was exported from the treatment planning system (TPS) and preprocessed using the MedicalDataHandler software, which was used for detailed dataset validation, uniform data resampling to an isotropic voxel resolution of 2 × 2 × 2 mm^3^, and conversion of CT images from Hounsfield Units (HU) to Relative Electron Densities (RED) using our institutional calibration curve data.^20^

### 2.3 Input encoding

The input for each plan was encoded into an 8‐channel tensor. The first channel contained the planning CT image, expressed in units of RED and normalized to the range [0, 1.0]. The second channel represented all OARs by assigning a unique random decimal value in the range (0, 1.0] to each OAR's physical location. The third channel encoded the Euclidean distance to the nearest isocenter, in units of meters. This feature provides spatial context that helps the network learn beam geometry patterns, particularly the characteristic dose peaks at beam entry and exit points. Without distance information, minor spatial misalignments in predicted beam entry and exit regions would incur steep loss penalties during training. In clinical practice, if the plan isocenter is not yet chosen, the PTV centroid can serve as a reasonable surrogate.

The fourth channel included plan‐specific PTVs, encoded in units of Gy/100. The fifth through seventh channels contained spatially encoded plan‐sum dosimetric goals (maximum, mean, and volumetric), which are created by spatially mapping goals to each corresponding structure's physical location. For maximum and mean goals, the goal dose value was assigned uniformly to all voxels within the structure. For volumetric goals (e.g., V20 Gy < 30%), the constraint dose was assigned only to the spared fraction of the structure's volume (e.g., 70%), prioritizing voxels most distal from the PTV(s) based on Euclidean distance. This method of dosimetric goal mapping is similar to the approach described in our previous work.^14^ The eighth channel encoded plan‐sum PTVs, also in units of Gy/100, representing the cumulative PTVs across the full course of treatment.

### 2.4 Model training

Both models were trained from scratch using randomly initialized weights and the AdamW optimizer with a fixed learning rate of 1 × 10^−4^. To accommodate GPU memory limitations, input data consisted of randomly sampled patches with dimensions 128 × 128 × 96 voxels. Training was performed with a batch size of 3, where each batch was composed of data from a single patient. Models were trained on an NVIDIA A100 GPU with 80 GB of VRAM, while inference for the test set was performed using an NVIDIA RTX 4090 GPU with 24 GB of VRAM.

While both models were exposed to all plans across training, they differed in their batch sampling strategy. For the single‐plan model, one plan was randomly selected per patient at each epoch, and three augmented samples were drawn from that plan to form a batch. Over the course of training, the single‐plan model observed all plans in the dataset, but each plan was sampled in isolation without concurrent access to related plans in the same treatment course. In contrast, the multi‐plan model included all available plans for a patient in each batch, providing simultaneous access to the full treatment course context. If fewer than three plans were available, additional samples were drawn at random from that patient's plans to reach the target batch size.

To ensure reproducibility across both model trainings, a fixed random seed was set. Training was run for a conservative 30 000 epochs to ensure convergence of both models, and model weights were saved at the epoch corresponding to the lowest validation loss.

#### 2.4.1 Loss function

Voxel‐wise loss functions—such as Mean Squared Error (MSE)—may excessively penalize minor spatial misalignments, negatively impacting training stability and model generalization. To mitigate this, we adopted a multi‐objective optimization strategy that simultaneously optimizes voxel‐wise accuracy and regional structural coherence by combining MSE with the Multi‐Scale Structural Similarity Index (MS‐SSIM). This approach encourages a balance in voxel‐wise accuracy with regional structural consistency at broader spatial scales.

The total loss function, L(θ), was defined as a two‐dimensional vector comprising the MSE and MS‐SSIM components, where θ represents the vector of trainable model parameters (Equation 1).

(1) $$L\;\left(\theta \right)=\left[L_{MSE}\left(\theta \right),L_{MS-SSIM}\left(\theta \right)\right]$$

The first component, $L_{MSE}$, quantifies voxel‐wise dose differences between the predicted (*P*) and ground‐truth label (*L*) dose distributions (Equation 2).

(2) $$L_{MSE}=\frac{1}{D\times H\times W}\;\sum_{d=1}^{D}\sum_{h=1}^{H}\sum_{w=1}^{W}{\left(P\left(d,h,w\right)-L\left(d,h,w\right)\right)}^{2}\;$$

The second component, $L_{MS-SSIM}$, is a perceptual loss that promotes structural similarity across multiple (*M*) scales (Equation 3).

(3) $$L_{MS-SSIM}=1-\frac{1}{M}\sum_{i=1}^{M}\left(SSIM_{i}{\left(P_{i},L_{i}\right)}^{\beta_{i}}\right)$$

Here, $\beta_{i}$ is the relative weight assigned to scale i, where $\sum_{i\;=1}^{M}\beta_{i}=1$. At each scale i, the prediction and ground truth volumes are smoothed with a Gaussian low‐pass filter and spatially downsampled by a factor of $2^{i-1}$. In the single‐scale case (i.e., *M* = 1), MS‐SSIM reduces to the standard SSIM formulation (Equation 4).

(4) $$SSIM\left(P,\;L\right)=\;\;\frac{\left(2\mu_{P}\mu_{L}+C_{1}\right)\left(2\sigma_{PL}+C_{2}\right)}{\left(\mu_{P}^{2}+\mu_{L}^{2}+C_{1}\right)\left(\sigma_{P}^{2}+\sigma_{L}^{2}+C_{2}\right)}$$

Here, $\mu_{P}$ and $\mu_{L}$ are local means, $\sigma_{P}^{2}$ and $\sigma_{L}^{2}$ are local variances, $\sigma_{PL}$ is the local covariance, and $C_{1}$ and $C_{2}$ are constants used to stabilize the denominator.

To ensure balanced optimization of these two distinct objectives, we implemented Jacobian descent via the TorchJD library.^21^ Jacobian descent treats the total loss as a vector‐valued function, unlike traditional approaches which combine scalar losses and compute a single gradient from their average. At each training iteration, the Jacobian matrix (J) of gradients is computed, which contains individual gradients of each loss component (Equation 5).

(5) $$J=\;\left[\begin{array}{c} \nabla_{\theta }L_{MSE}\left(\theta \right)\\ \nabla_{\theta }L_{MS-SSIM}\left(\theta \right) \end{array}\right]$$

Simply averaging gradients can lead to updates that benefit one objective at the expense of another, so we instead used the TorchJD implementation of the Unconflicting Projection of Gradients aggregator ($A_{\mathrm{UPGrad}}$). This aggregator projects each gradient onto the dual cone defined by all other gradients before averaging them, ensuring that the resulting parameter updates are beneficial—or at least non‐harmful—to both objectives, thereby improving training stability and balance of task importance in multi‐objective optimization.

#### 2.4.2 Data augmentation

To improve model generalization and reduce overfitting, we employed data augmentation techniques during training. Broadly, overfitting occurs when a model memorizes training data too precisely and therefore has limited ability to generalize to the new cases (e.g., poor performance on validation/test subsets). Transformations were applied stochastically and independently to each training sample, and multiple transformations could be applied concurrently.

Patch location was randomized by sampling a center from a multivariate Gaussian distribution centered at the plan isocenter, with standard deviations set to half the size of each corresponding spatial dimension. From the sampled center, a 3D patch of size 128 × 128 × 96 voxels were extracted. If any volume dimension was smaller than the patch size, padding was applied to maintain consistent input shape.

Spatial transformations included a 25% chance of random rotation by 0°, 90°, 180°, or 270° (in the [y, x] plane), a 50% chance of mirroring along the x‐axis, and a 50% chance of flipping along the z‐axis. These augmentations, combined with randomized patch sampling, encouraged spatial invariance.

To encourage scale invariance and support learning across variable fractionation schemes, dose distributions were rescaled to a random integer number of fractions between zero and the prescribed number. Correspondingly, the plan‐sum dosimetric goals and plan‐sum dose label were rescaled to reflect the sum of all fraction‐scaled plans for that patient. For example, if plan #1 was scaled down from 20 fractions to 5 fractions, and plan #2 from 15 fractions to 8 fractions, the plan‐sum data was scaled down from 35 fractions to 13 fractions. This rescaling, as with all augmentation in this section, was applied only during training. Validation and test inference both operated on un‐augmented data at the clinical prescription. Model checkpoints were selected on that data, and all reported test metrics were computed on it. Therefore, this augmentation schedule is a training‐dynamics knob that may improve the model's interpolation across prescription levels under‐represented in the training distribution, potentially supporting robust performance at clinical deployment.

To encourage task independence and minimize overfitting to plan‐sum input data, the plan‐sum PTV and the corresponding plan‐sum dose label were randomly zeroed out with a 25% probability.

OARs were encoded by mapping a random unique decimal value to each of their physical locations. To prevent the model from overfitting to fixed OAR values, we randomly re‐assigned these values with a 50% probability, thereby encouraging the network to focus on spatial relationships rather than learning static values.

To minimize overfitting to specific plan‐sum dosimetric goal values, each goal channel had a 50% chance of omitting one randomly selected goal, a 50% chance of smoothing the goals with a Gaussian filter, a 50% chance of adding Gaussian noise, and a 50% chance of scaling goal intensities by a random factor in the range [0.8, 1.2]. Without these augmentations, the model risks overfitting to specific goal values encountered during training rather than learning generalizable dose allocation patterns. These augmentations encourage the model to interpret goals as approximate guidance in‐context with the other input channels, rather than learning goals as fixed mappings to memorize.

### 2.5 Evaluation metrics

To assess the performance of our dose prediction models, we predicted dose distributions for all plans in the test set and then resampled each of them to their original voxel spacing. We evaluated the difference between these dose predictions and the ground truth dose distributions with several quantitative metrics. Importantly, all metrics excluded voxels that are outside the dose calculation volume to prevent bias from zero‐valued regions.

Mean Absolute Error (MAE) measures the average absolute difference in dose (Gy) between the predicted and ground truth distributions. We selected MAE for evaluation rather than MSE because MAE provides direct clinical interpretability in units of Gy, whereas MSE disproportionately weights large errors and yields values without intuitive physical meaning. MSE remains appropriate for training due to its smooth gradients and suitability for optimization. However, because MAE is scale‐dependent, it may not effectively reflect performance across datasets with wide variability in prescription dose values. To address this limitation, we normalized MAE by the prescription dose (Rx) and expressed it as a percentage, yielding a scale‐invariant metric that we refer to as MAE/Rx (Equation 6).

(6) $$\begin{array}{c} \frac{MAE}{Rx}\left(\%\right)=\frac{100}{Rx\times D\times H\times W}\;\sum_{d=1}^{D}\sum_{h=1}^{H}\sum_{w=1}^{W}\left|P\left(d,h,w\right)-L\left(d,h,w\right)\right| \end{array}$$

This normalization approach was similarly applied to Root Mean Square Error (RMSE) to yield RMSE/Rx (%) (Equation 7).

(7) $$\begin{array}{cc} & \frac{\mathrm{RMSE}}{\mathrm{Rx}}\left(\%\right) \end{array}=\frac{100}{\mathrm{Rx}}\;\sqrt{\frac{1}{D\times H\times W}\sum_{d=1}^{D}\sum_{h=1}^{H}\sum_{w=1}^{W}P\left(d,h,w\right)-L{\left(d,h,w\right)}^{2}}$$

To assess organ‐specific accuracy, we computed the OAR Mean‐Dose Error ($MDE_{OARS}$), defined as the absolute difference between the predicted and ground truth mean dose values for each OAR. These were averaged over all OARs and expressed as a percentage of the prescription dose, producing a scale‐invariant form (Equation 8).

(8) $$\frac{MDE_{OARS}}{\mathrm{Rx}}\left(\%\right)=\frac{100}{\mathrm{Rx}\times N_{OAR}}\;\sum_{i=1}^{N_{OAR}}\left|P_{mean}^{\left(i\right)}-L_{mean}^{\left(i\right)}\right|$$

We evaluated the dose‐volume histogram error (DVHE) for the body and OARs using a binned histogram difference method. First, both the predicted and ground truth dose distributions were expressed as a percentage of the prescription dose. A total of 110 bins were defined at 1% intervals from 0% to 110% of the prescription dose. Cumulative DVHs were computed for both the prediction and ground truth dose distributions. The absolute difference between cumulative bin values was then averaged across all bins. $DVHE_{OARS}$ is reported as the average over all OARs (Equation 9), and $DVHE_{BODY}$ is only for the body.

(9) $$\begin{array}{c} DVHE_{OARS}\;\left(\%\right)=\frac{1}{N_{OARS}}\sum_{i=1}^{N_{OARS}}\left(\frac{1}{B}\sum_{b=1}^{B}\left|D_{pred}^{\left(i\right)}\left(b\right)-D_{label}^{\left(i\right)}\left(b\right)\right|\right) \end{array}$$

Lastly, we quantified dose pattern errors across spatial scales between the predicted and ground truth dose distributions using the Frequency Histogram Error (FHE). This metric is spatially invariant, as it captures differences in dose pattern structure across spatial frequencies rather than at specific voxel locations. Radial frequency bins were defined by converting integer voxel intervals (ranging from 2 to 300 voxels) to spatial frequencies ($mm^{-1}$) based on the plan's voxel spacing. Each bin therefore corresponds to a spatial scale, where smaller voxel intervals reflect finer details (higher frequencies) and larger intervals reflect coarser features (lower frequencies). Dose distributions were normalized by prescription dose. A 3D Fast Fourier Transform (FFT) was applied to each dose distribution, and the resulting magnitude spectrum was flattened to 1D. We calculated the mean FFT magnitude, $\bar{A}$, within each radial frequency bin. FHE was defined as the mean relative absolute error between the predicted and ground truth frequency histograms across all B bins, with $\varepsilon =10^{-6}$ to avoid division by zero (Equation 10).

(10) $$FHE\;\left(\%\right)=\frac{100}{B}\;\sum_{b=1}^{B}\left|\frac{{\bar{A}}_{pred}\left(b\right)-{\bar{A}}_{label}\left(b\right)}{{\bar{A}}_{label}\left(b\right)+\varepsilon }\right|$$

## 3 RESULTS

Both the single‐plan model and the multi‐plan model were trained for 30 000 epochs, and model weights were saved at the epoch with the lowest validation loss. These final model weights were used to predict dose for the test set, which consisted of 41 plans and 20 plan‐sums across 20 patients. Since the single‐plan model does not explicitly predict plan‐sum doses, plan‐sums were computed by summing the model's per‐plan predictions. In contrast, the multi‐plan model predicts a plan‐sum dose alongside each plan dose; these plan‐sum dose predictions were averaged to obtain a single plan‐sum dose per patient for evaluation. To ensure consistent and clinically meaningful evaluation, doses were resampled to original spatial resolution and scaled such that the volume of the PTV receiving prescription dose matched that of the corresponding clinical plan. Statistical significance for all metric comparisons was assessed using the two‐tailed Wilcoxon signed‐rank test, with False Discovery Rate (FDR) correction applied to account for multiple comparisons.

### 3.1 Plan‐level dose evaluation

Quantitative comparison of plan‐specific model performance on the test set is shown in Table 2. Across most metrics, the multi‐plan model's results were superior to those of the single‐plan model and their differences were statistically significant. This includes reductions in voxel‐wise metrics such as MAE/Rx (1.244 ***± *** 0.151% vs. 1.650 ***± *** 0.172%, *p* < 0.001) and RMSE/Rx (3.259 ***± *** 0.297% vs. 3.753 ***± *** 0.334%, *p* < 0.001). Organ‐specific metrics also demonstrated consistent reductions in error, including lower mean‐dose error ($MDE_{OARS}$/Rx; 4.522 ± 0.606% vs. 6.228 ± 0.817%, *p* < 0.001) and lower DVH error ($DVHE_{OARS}$; 4.695 ± 0.554% vs. 6.230 ± 0.724%, *p* < 0.001). In addition, the multi‐plan model's dose predictions exhibited lower error in dose patterns across spatial frequency bands (FHE; 10.751 ± 2.568% vs. 13.314 ± 3.183%, *p* < 0.001), and greater structural similarity (SSIM; 0.964 ± 0.005% vs. 0.944 ± 0.009%, *p* < 0.001). However, we note that the differences in OAR and PTV $D_{MAX}$ were not statistically significant. We attribute this to maximum dose constraints being largely plan‐invariant, whereas volume‐based metrics may have a greater dependence on PTV‐OAR spatial relationships that can shift substantially between initial and boost volumes as PTV size decreases.

**TABLE 2:** Plan‐specific dose metric comparison to assess model performance on the test set.

Metric (unit)	Single‐Plan Model	Multi‐Plan Model	*p*‐value
$DVHE_{BODY}$ (%)	**0.858 ± 0.122**	***0.484 ± 0.080***	3.49e‐11***
$DVHE_{OARS}$ (%)	**6.230 ± 0.724**	***4.695 ± 0.554***	5.10e‐08***
FHE (%)	**13.314 ± 3.183**	***10.751 ± 2.568***	4.95e‐06***
MAE (Gy)	**0.521 ± 0.101**	***0.399 ± 0.083***	1.46e‐11***
MAE /Rx (%)	**1.650 ± 0.172**	***1.244 ± 0.151***	1.46e‐11***
$MDE_{OARS}$ (Gy)	**2.001 ± 0.378**	***1.468 ± 0.297***	4.83e‐08***
$MDE_{OARS}/Rx$ (%)	**6.228 ± 0.817**	***4.522 ± 0.606***	4.01e‐08***
$OAR\;D_{MAX}\;Error$ (Gy)	2.414 ± 0.791	*2.202 ± 0.821*	1.64e‐01
$OAR\;D_{MAX}\;Error/Rx$ (%)	8.153 ± 2.448	*7.413 ± 2.525*	1.64e‐01
$PTV\;D_{MAX}$ (Gy)	***0.713 ± 0.194***	**1.29 ± 0.265**	1.80e‐05***
$PTV\;D_{MAX}/Rx$ (%)	***2.301 ± 0.502***	**4.704 ± 1.074**	3.76e‐06***
RMSE (Gy)	**1.192 ± 0.217**	***1.029 ± 0.180***	2.23e‐09***
RMSE /Rx (%)	**3.753 ± 0.334**	***3.259 ± 0.297***	1.41e‐09***
SSIM	**0.944 ± 0.009**	***0.964 ± 0.005***	7.96e‐08***

*Notes*: Values are reported as mean ± 95% confidence interval. Bold indicates statistically significant difference (*p* < 0.05). Italics denote the better performing model.

Abbreviations: DVHE, dose‐volume histogram error; FHE, Fourier Histogram Error; MAE, Mean Absolute Error; MDE, Mean‐Dose Error; OAR, organ at risk; RMSE, Root Mean Square Error; Rx, prescription dose; SSIM, structural similarity index measure.

*p < 0.05, **p < 0.01, ***p < 0.001.

### 3.2 Plan‐sum dose evaluation

Comparison of plan‐sum‐specific model performance on the test set is summarized in Table 3. Similar to plan‐level results, the multi‐plan model achieved superior results most metrics compared to the single‐plan model. Specifically, the multi‐plan model showed lower MAE/Rx (1.146 ± 0.174% vs. 1.525 ± 0.188%, *p* < 0.001) and RMSE/Rx (2.791 ± 0.324% vs. 3.310 ± 0.374%, *p* < 0.001). Additionally, the multi‐plan model exhibited lower organ‐specific errors in mean‐doses ($MDE_{OARS}$/Rx; 3.973 ± 0.694% vs. 6.091 ± 0.900%, *p* < 0.001) and DVHs ($DVHE_{OARS}$; 4.181 ± 0.641% vs. 5.963 ± 0.803%, *p* < 0.001). Although the multi‐plan model showed a reduction in FHR, this difference was not statistically significant (FHE; 9.640 ± 3.343% vs. 11.164 ± 4.342%, *p* = 0.095). Relatedly, the structural similarity was moderately improved for the multi‐plan model (SSIM; 0.972 ± 0.006% vs. 0.960 ± 0.006%, *p* < 0.001).

**TABLE 3:** Plan‐Sum‐specific dose metric comparison to assess model performance on the test set.

Metric (unit)	Single‐Plan Model	Multi‐Plan Model	*p*‐value
$DVHE_{BODY}$ (%)	**0.781 ± 0.155**	***0.475 ± 0.122***	5.09e‐06***
$DVHE_{OARS}$ (%)	**5.963 ± 0.803**	***4.181 ± 0.641***	5.09e‐06***
FHE (%)	11.164 ± 4.342	*9.640 ± 3.343*	9.48e‐02
MAE (Gy)	**0.958 ± 0.119**	***0.719 ± 0.106***	5.09e‐06***
MAE /Rx (%)	**1.525 ± 0.188**	***1.146 ± 0.174***	5.09e‐06***
$MDE_{OARS}$ (Gy)	**3.786 ± 0.451**	***2.474 ± 0.390***	5.09e‐06***
$MDE_{OARS}/Rx$ (%)	**6.091 ± 0.900**	***3.973 ± 0.694***	5.09e‐06***
$OAR\;D_{MAX}\;Error$ (Gy)	3.994 ± 1.717	*3.558 ± 1.664*	1.65e‐01
$OAR\;D_{MAX}\;Error/Rx$ (%)	6.158 ± 2.603	*5.462 ± 2.523*	1.64e‐01
$PTV\;D_{MAX}$ (Gy)	*1.207 ± 0.435*	1.467 ± 0.531	4.98e‐01
$PTV\;D_{MAX}/Rx$ (%)	*1.938 ± 0.668*	2.353 ± 0.826	4.98e‐01
RMSE (Gy)	**2.089 ± 0.251**	***1.765 ± 0.219***	8.42e‐06***
RMSE /Rx (%)	**3.310 ± 0.374**	***2.791 ± 0.324***	8.42e‐06***
SSIM	**0.960 ± 0.006**	***0.972 ± 0.006***	1.30e‐04***

*Notes*: Values are reported as mean ± 95% confidence interval. Bold indicates statistically significant difference (*p* < 0.05). Italics denote the better performing model.

Abbreviations: DVHE, dose‐volume histogram error; FHE, Fourier Histogram Error; MAE, Mean Absolute Error; MDE, Mean‐Dose Error; OAR, organ at risk; RMSE, Root Mean Square Error; Rx, prescription dose; SSIM, structural similarity index measure.

*p < 0.05, **p < 0.01, ***p < 0.001.

### 3.3 Overall error analysis

**FIGURE 3:**
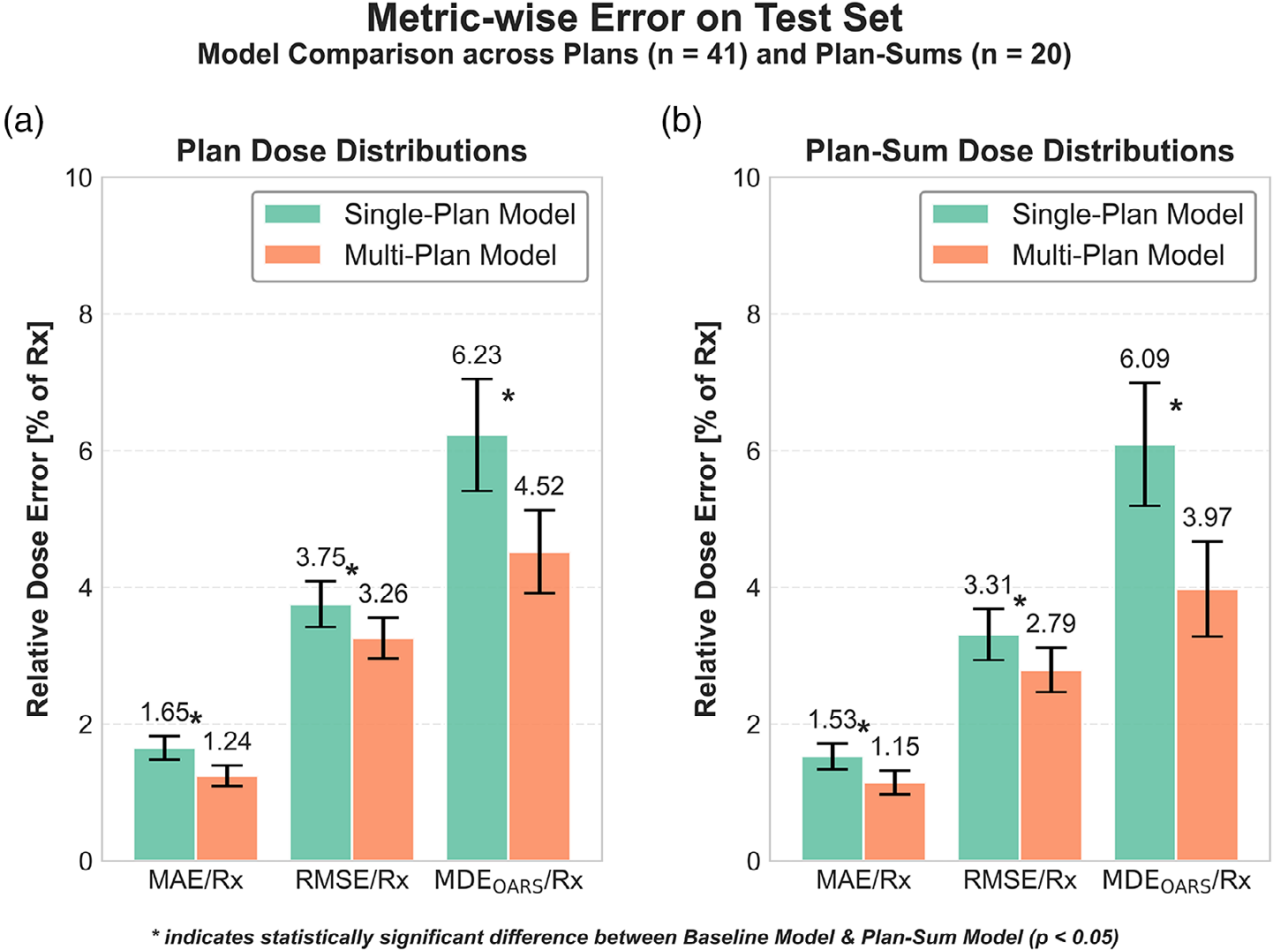
Test set performance comparison between the single‐plan model and multi‐plan model across key metrics: Mean Absolute Error as a percentage of prescription dose (MAE/Rx), Root Mean Square Error as a percentage of prescription dose (RMSE/Rx), and Mean‐Dose Error averaged across organs at risk (OARs) as a percentage of prescription dose ($MDE_{OARS}$/Rx). Error bars indicate 95% confidence intervals. Asterisks (*) indicate statistically significant differences between models (*p* < 0.05). Single‐plan model results are shown in green‐cyan; multi‐plan model results are shown in red‐orange. (a) Plan‐level dose distributions (*n* = 41). (b) Plan‐sum dose distributions (*n* = 20).

Figure 3 presents a comparison of model performance for both plan‐level (Figure 3a) and plan‐sum (Figure 3b) dose distributions across three important metrics: MAE/Rx, RMSE/Rx, and OAR Mean‐Dose Error ($MDE_{OARS}$/Rx). In all six comparisons, the multi‐plan model demonstrated statistically significant differences compared to the single‐plan model, with consistently lower mean errors and narrower 95% confidence intervals.

**FIGURE 4:**
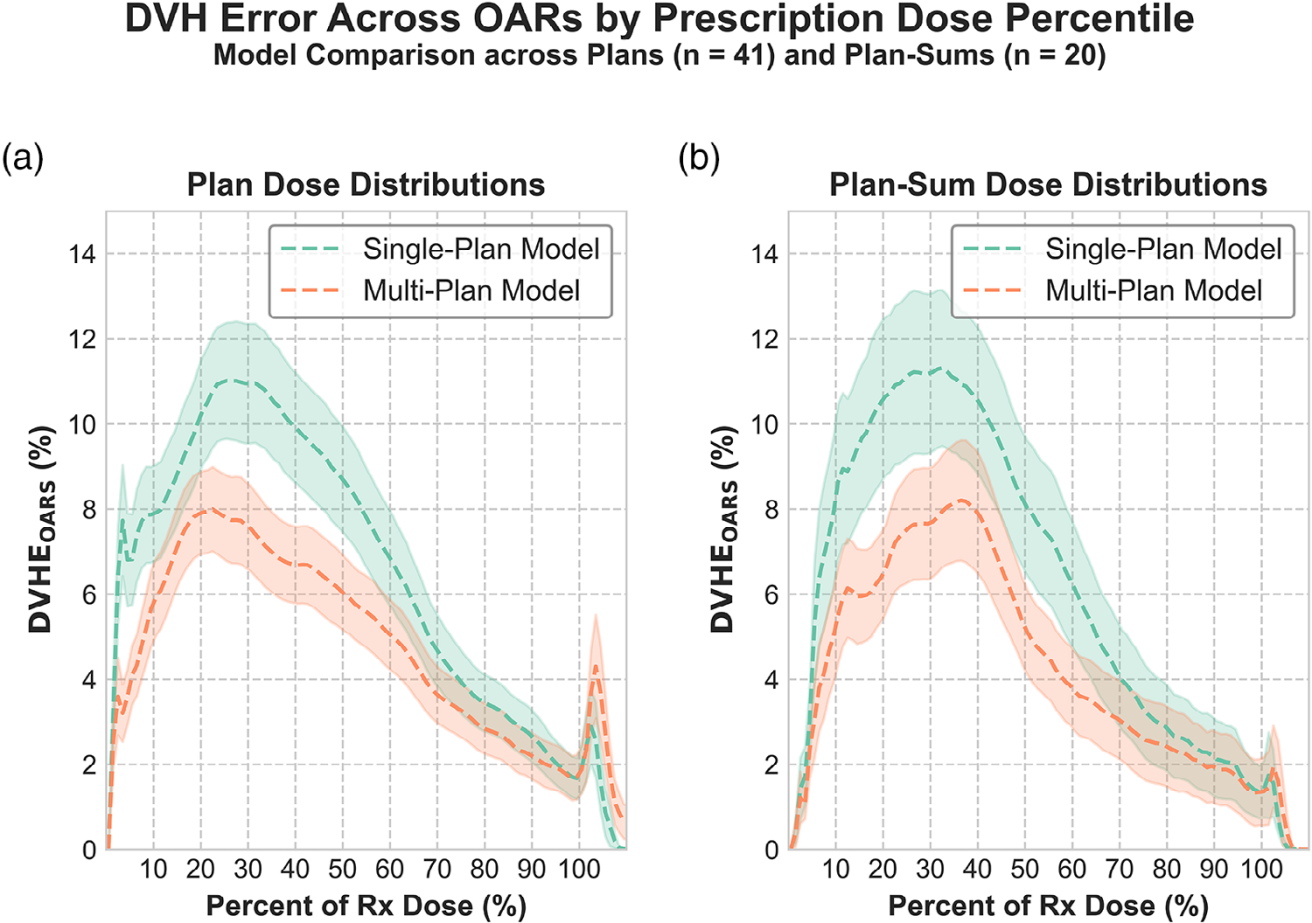
Dose‐volume histogram (DVH) error by prescription dose percentile, averaged across organs at risk (OARs). For each test case, DVH error (DVHE) was first computed per OAR and binned by percent of prescription dose. DVHE represents the absolute difference in cumulative volume (%) between predicted and ground truth DVH curves; for example, 8% error at V20 indicates the predicted volume is within ± 8 percentage points of ground truth. Because DVHE is computed independently at each dose bin, errors may vary in direction across the DVH curve (e.g., V20 underestimated while V25 overestimated), reflecting sensitivity to spatial alignment rather than systematic bias. Bin errors were averaged across OARs within each patient ($DVHE_{OARS}$), and then averaged across the test set. Shaded regions indicate 95% confidence intervals. The multi‐plan model (red‐orange, dashed) consistently demonstrated lower error than the single‐plan model (green‐cyan, dashed) across a majority of the dose range. (a) Plan‐level dose distributions (*n* = 41). (b) Plan‐sum dose distributions (*n* = 20).

To evaluate dosimetric agreement in more clinically interpretable terms, Figure 4 visualizes OAR DVH error relative to prescription dose ($DVHE_{OARS}$). The multi‐plan model exhibited lower error and narrower 95% confidence intervals than the single‐plan model across most bins, especially within the 20%–70% Rx range where dose conformity and OAR sparing are most often relevant.

**FIGURE 5:**
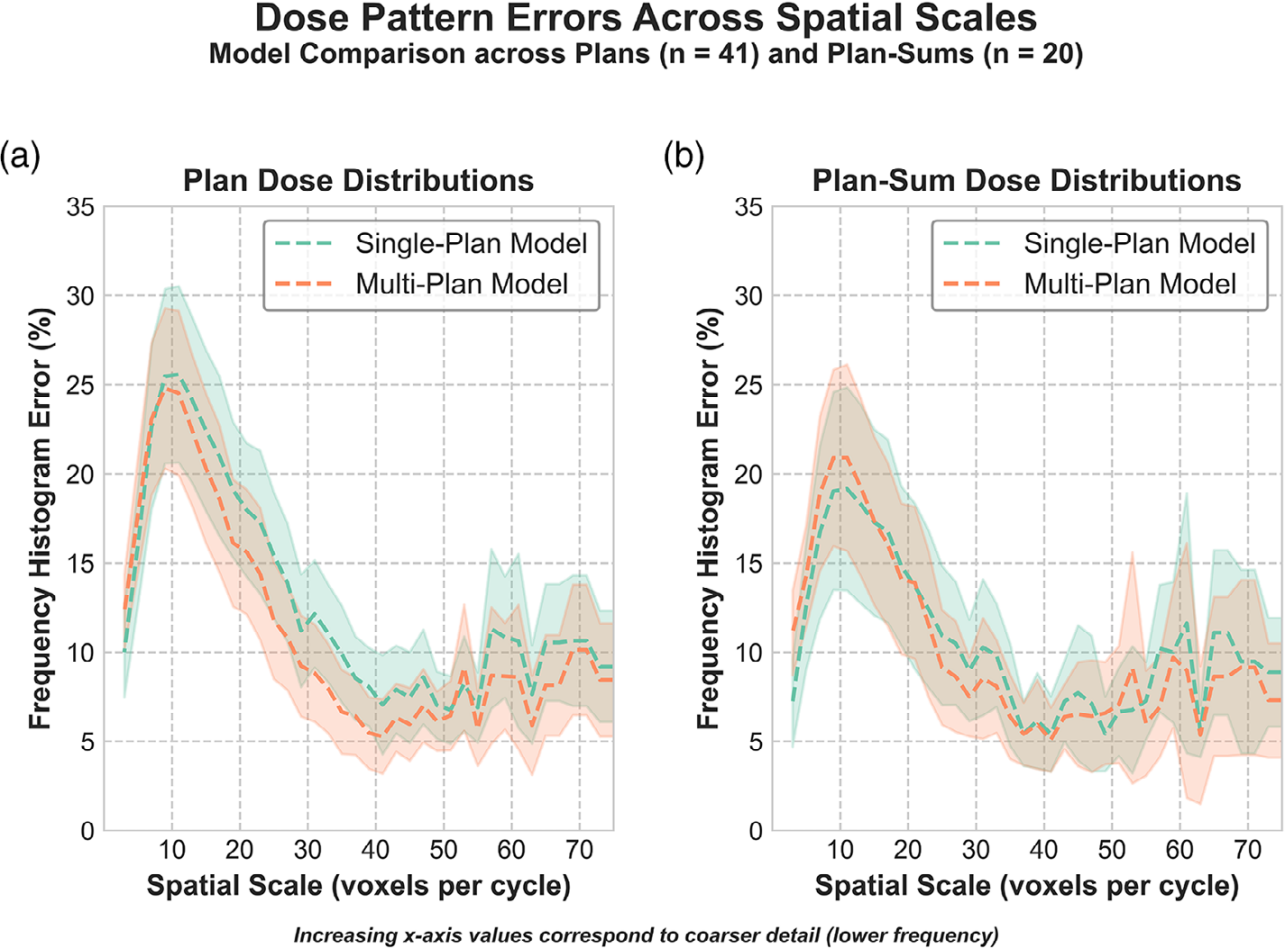
Frequency Histogram Error (FHE) across spatial scales. FHE measures how well predicted dose patterns match ground truth by comparing their spatial frequency content. The x‐axis represents spatial scale in voxels per cycle: smaller values (left) capture fine details like sharp dose gradients, while larger values (right) capture broader features like overall dose shape. Unlike Dice coefficients, which only evaluate overlap at selected dose levels, FHE assesses pattern agreement across all scales simultaneously. Each curve shows the mean FHE averaged across test cases; shaded regions indicate 95% confidence intervals. Lower values indicate better agreement. The multi‐plan model (red‐orange, dashed) demonstrates consistently lower errors than the single‐plan model (green‐cyan, dashed) across most spatial scales in plan dose distributions, and comparable performance in plan‐sum dose distributions. (a) Plan‐level dose distributions (*n* = 41). (b) Plan‐sum dose distributions (*n* = 20).

Dose distributions from each model were evaluated across spatial scales using FHE, which quantifies differences in spatial dose patterns independent of voxel location. As shown in Figure 5, the multi‐plan model exhibited consistently lower error across most spatial scales in plan‐level dose distributions (Figure 5a) and comparable error in plan‐sum dose distributions (Figure 5b). However, the differences in spatial agreement were more pronounced for plan‐level dose distributions than plan‐sums.

To assess whether prediction errors exhibited systematic directional bias, we computed signed mean dose error across OARs (Table 4). The baseline, single‐plan model showed systematic overestimation at both plan‐level (75.4 ± 5.5% of OAR doses overestimated, mean signed error: +1.20 ± 0.40 Gy) and plan‐sum level (73.1 ± 8.6% overestimated, +2.46 ± 0.80 Gy). In contrast, the proposed multi‐plan model showed no systematic bias, with errors distributed nearly equally between over‐ and underestimation (plan‐level: 49.5 ± 7.3% overestimated, +0.18 ± 0.37 Gy; plan‐sum: 47.6 ± 9.1% overestimated, +0.21 ± 0.74 Gy). The differences in overestimation percentage were statistically significant for both plan‐level (*p* < 0.001) and plan‐sum (*p* < 0.001) predictions.

**TABLE 4:** Signed mean dose error analysis across OARs.

Metric (unit)	Comparison Type	Single‐Plan Model	Multi‐Plan Model	*p*‐value
Signed Error (Gy)	Plan	**1.20 ± 0.40**	***0.18 ± 0.37***	1.23e‐06***
Signed Error (Gy)	Plan‐Sum	**2.46 ± 0.80**	***0.21 ± 0.74***	9.44e‐05***
Signed Error/Rx (%)	Plan	**3.40 ± 1.13**	***0.58 ± 1.00***	4.30e‐06***
Signed Error/Rx (%)	Plan‐Sum	**3.59 ± 1.29**	***0.48 ± 1.14***	1.02e‐03**
Overestimate (%)	Plan	**75.4 ± 5.5**	***49.5 ± 7.3***	4.30e‐06***
Overestimate (%)	Plan‐Sum	**73.1 ± 8.6**	***47.6 ± 9.1***	6.21e‐05***
Underestimate (%)	Plan	**24.6 ± 5.5**	***50.5 ± 7.3***	4.30e‐06***
Underestimate (%)	Plan‐Sum	**26.9 ± 8.6**	***52.4 ± 9.1***	3.38e‐04***

*Notes*: Positive signed error indicates overestimation (predicted > ground truth). Values are reported as mean ± 95% confidence interval. Bold indicates statistically significant difference (*p* < 0.05). Italics denote the better‐performing model.

Abbreviations: OAR, organ at risk; Rx, prescription dose.

*p < 0.05, **p < 0.01, ***p < 0.001.

### 3.4 Qualitative comparison

**FIGURE 6:**
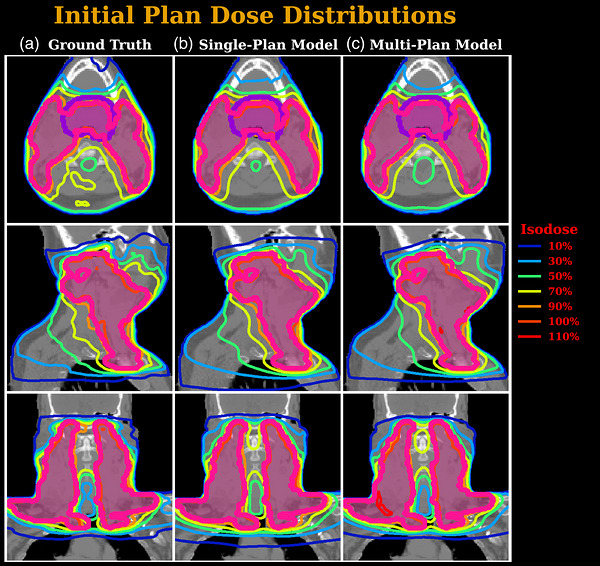
Dose distributions for the initial plan in a Head and Neck (H&N) treatment course delivering a simultaneous boost of 32 and 40 Gy over 20 fractions, overlaid on the CT image across axial, sagittal, and coronal views (top to bottom). Pink and purple filled contours indicate the planning target volumes (PTVs). Panels (a)–(c) represent dose for the clinically approved ground truth, single‐plan model, and multi‐plan model, respectively. Notably, the multi‐plan model demonstrates improved dose accuracy in the oral cavity (axial views), the inferior boundary (sagittal and coronal views), and the larynx and parotids (coronal views).

**FIGURE 7:**
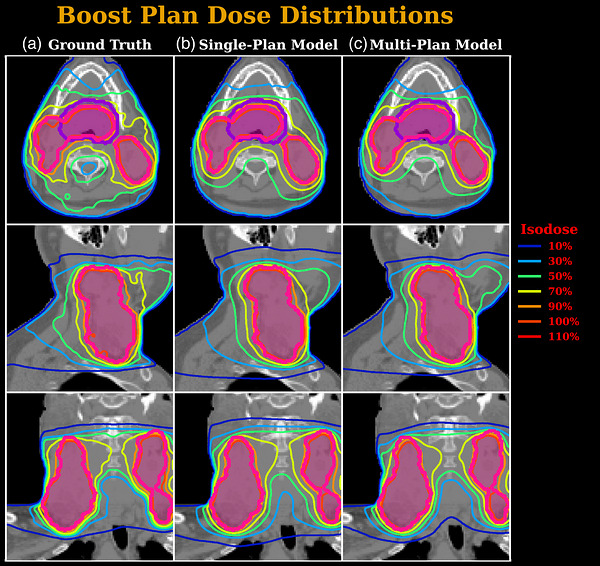
Dose distributions for the boost plan in a Head and Neck (H&N) treatment course delivering a simultaneous boost of 24 and 30 Gy over 15 fractions, overlaid on the CT image across axial, sagittal, and coronal views (top to bottom). Pink and purple filled contours indicate the planning target volumes (PTVs). Panels (a)–(c) represent dose for the clinically approved ground truth, single‐plan model, and multi‐plan model, respectively. The most distinguishable dose difference appears in the larynx region, as seen in the coronal views.

**FIGURE 8:**
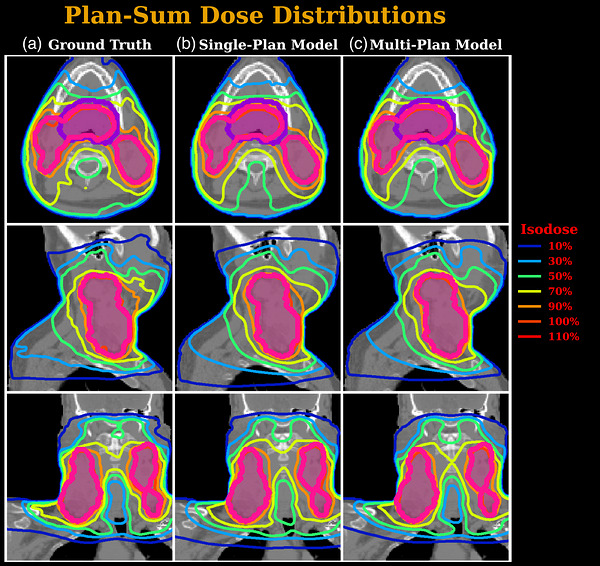
Dose distributions for the plan‐sum in a Head and Neck (H&N) treatment course delivering a total of 56 and 70 Gy over 35 fractions, overlaid on the CT image across axial, sagittal, and coronal views (top to bottom). Pink and purple filled contours indicate the planning target volumes (PTVs). Panels (a)–(c) represent dose for the clinically approved ground truth, single‐plan model, and multi‐plan model, respectively. The greatest dose differences are observed in the larynx and parotids, which are visible in the coronal views.

**FIGURE 9:**
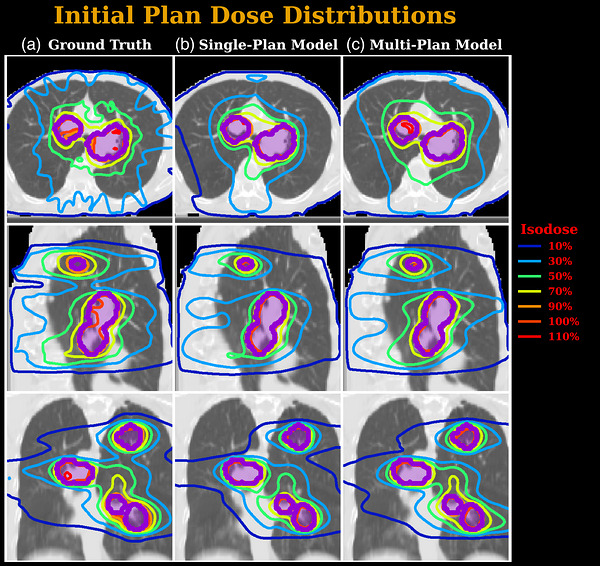
Dose distributions for the initial plan in a lung treatment course delivering 60 Gy over 30 fractions, overlaid on the CT image across axial, sagittal, and coronal views (top to bottom). The purple filled contour indicates the planning target volume (PTV). Panels (a)–(c) represent dose for the clinically approved ground truth, single‐plan model, and multi‐plan model, respectively. In the axial views, the multi‐plan model exhibits a dose gradient more consistent with the clinical ground truth, whereas the single‐plan model shows an abrupt drop‐off from 42 to 24 Gy. Given that only one lung patient was included in the training set, this result suggests that the multi‐plan model may generalize better to sparsely represented cases than the single‐plan model.

**FIGURE 10:**
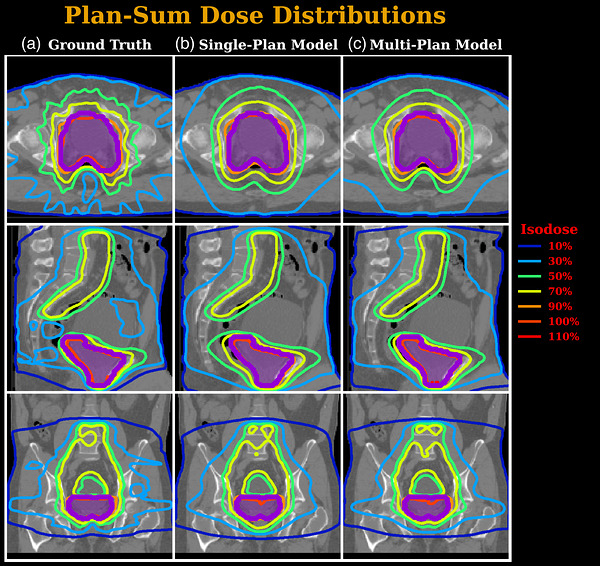
Dose distributions for the plan‐sum in a prostate treatment course delivering a total of 64.8 Gy over 36 fractions, overlaid on the CT image across axial, sagittal, and coronal views (top to bottom). The purple filled contour indicates the planning target volumes (PTV). Panels (a)–(c) represent dose for the clinically approved ground truth, single‐plan model, and multi‐plan model, respectively. Improved dose accuracy is demonstrated in the rectum (axial and sagittal views).

A comparison of single‐plan model and multi‐plan model performance is qualitatively evaluated in Figures 6, 7, 8, 9, 10, spanning multiple disease sites and fractionation schemes. Each figure shows the clinically delivered ground truth dose distribution in the first column, the single‐plan model dose distribution in the second column, and the multi‐plan model dose distribution in the third column. All dose distributions are overlaid on CT images across axial, sagittal, and coronal view rows, with PTV contours delineated.

A complete H&N treatment course is shown in Figures 6, 7, 8, covering the initial plan, boost plan, and plan‐sum dose distributions, respectively. In Figure 6, the multi‐plan model dose demonstrates improved accuracy in multiple sub‐regions, including the oral cavity (axial view), the sharpness of the inferior boundary (sagittal view), and the larynx and parotids (coronal view). Similarly, the boost plan in Figure 7 reflects distinguishable improvement in the larynx (coronal view), where the single‐plan model tends to underestimate dose gradient sharpness around OARs. DVH comparisons are provided in Appendix Figures a1 and a3, respectively. Dose difference comparisons are provided in Appendix Figures a2 and a4, respectively.

The H&N plan sum in Figure 8 (DVH and dose difference comparisons provided in Appendix Figures a5 and a6, respectively) is consistent with the composite findings from the individual plans. In comparing the multi‐plan model with the single‐plan model, modest improvement is observed in the oral cavity (axial) and parotids (coronal), with more pronounced improvement in the larynx (coronal) and again in the inferior boundary of the dose distribution (sagittal and coronal).

To aid in visual assessment of potential model generalization across anatomical sites, Figure 9 (DVH and dose difference comparisons provided in Appendix Figures a7 and a8, respectively) shows dose distributions for a lung treatment plan. In the axial views, the multi‐plan model captures a more accurate dose gradient outside the PTV, particularly in the transition from 42 to 24 Gy, whereas the single‐plan model exhibits a dose drop‐off that is too rapid. Notably, this study included data from only two lung patients, one in training and one in testing. This single test case may illustrate stronger generalization with sparsely represented cases by the multi‐plan model than the single‐plan model, though more evidence is needed across a larger cohort to draw definitive conclusions.

Finally, Figure 10 (DVH and dose difference comparisons provided in Appendix Figures a9 and a10, respectively) illustrates plan‐sum dose distributions for a prostate case. In the axial view, the single‐plan model dose appears overly smooth and isotropic compared to the ground truth, whereas the multi‐plan model better captures sharper gradients consistent with beam entry and exit paths. Similar differences are seen in the inferior coronal views, where the multi‐plan model dose exhibits sharper lateral peaks than that of the single‐plan model. Additionally, the multi‐plan model dose shows improved accuracy in rectum sparing in the axial and sagittal views.

## 4 DISCUSSION

Our study introduces a multi‐plan deep learning framework for dose prediction specifically designed for sequential boost radiotherapy (RT). The key innovation lies in its ability to process and predict doses for individual plans and the cumulative plan‐sum simultaneously, providing the network with a global view of the entire treatment course. We found that this approach significantly outperforms a conventional single‐plan network, yielding more accurate dose predictions for both the distinct treatment phases and the total delivered dose. Critically, the architectural choices and input strategies employed—consolidated OAR representations, spatially encoded goals, and plan‐sum data—lay the groundwork for a universal dose prediction model, aiming for applicability to any disease site, fractionation scheme, or number of plans in a course.

The superior performance of the multi‐plan model was demonstrated quantitatively across several metrics, showing statistically significant improvements in voxel‐wise accuracy (lower MAE/Rx), organ‐specific DVH accuracy (lower $DVHE_{OARS}$), and structural similarity (greater SSIM) compared to the single‐plan baseline (see Tables 2, 3 and Figure 3). Qualitatively, dose distributions from the multi‐plan model exhibited closer resemblance to the ground truth clinical plans, notably producing sharper dose gradients near critical OARs (see Figures 6, 7, 8, 9, 10).

From a clinical perspective, the enhanced organ‐specific DVH accuracy (lower $DVHE_{OARS}$) is particularly meaningful because it directly reflects adherence to clinical organ‐sparing intent (see Figure 4). The primary application of dose prediction models in clinical workflows is to assist planners and physicians in pre‐plan guidance, enabling pre‐emptive identification of and discussion about achievable OAR doses and potential organ‐sparing trade‐offs.

Conversely, voxel‐wise metrics like MAE and RMSE are challenging to interpret due to their sensitivity to minor spatial misalignments. Our model does not make assumptions about beam templates or geometry, and the training data includes plans with varying beam configurations. The model learns to predict achievable dose distributions agnostic to specific beam arrangements. Since DVH metrics are insensitive to spatial rearrangement, two dose distributions achieving identical DVH metrics and target coverage may exhibit different spatial patterns depending on beam arrangement, causing voxel‐level metrics to penalize predictions that are clinically acceptable but geometrically dissimilar.

The typical application of dose prediction works is extraction of DVH‐based objectives rather than direct voxel‐level dose delivery. In practice, dose mimicking is performed by computing DVH curves from the predicted dose, from which optimization constraints (e.g., PTV D95, OAR mean, OAR volumetric sparing) can be extracted and applied in the TPS as a KBP‐style prior that seeds the initial objective list before the first optimization. Cao et al. prospectively demonstrated that plans generated with dose prediction guidance consistently improved OAR sparing compared to standard plans, highlighting the clinical utility of this workflow.^11^ Incorporating beam parameters would address voxel‐level discrepancies but presents significant challenges: variable beam and control point counts across plans, non‐trivial cross‐modality feature fusion, and the fact that beam configuration is determined iteratively during planning rather than known a priori. Accordingly, we emphasize metrics less sensitive to voxel‐level discrepancies, including $DVHE_{OARS}$, $MDE_{OARS}$, and SSIM.

Our analysis of dose pattern errors across spatial scales revealed that the multi‐plan model's advantage was most pronounced for individual plan dose predictions, though less pronounced for cumulative plan‐sum predictions (see Figure 5). This distinction reflects that individual plans exhibit greater spatial complexity and dose heterogeneity, whereas cumulative dose distributions become smoother when multiple plans are summed. Furthermore, the evaluation of multi‐plan model predictions involved averaging multiple plan‐sum predictions per patient, which may dampen fine‐grained spatial differences. However, the superiority at the individual plan level suggests that access to the full treatment context, including all target volumes and their spatial relationships, allows the model to better learn how dose should be preferentially allocated between PTVs and OARs for each individual plan when multiple plans are contributing to the same regions.

While single‐plan, site‐specific models are relatively common in clinical practice, they are fundamentally limited for sequential boost RT because they lack full treatment course context. It is also challenging, impractical, and inefficient to maintain multiple niche models for each disease site or fractionation scheme. Our multi‐plan framework addresses these limitations through three essential pre‐processing tasks: (1) consolidating multiple OARs into a single‐channel decimal‐value representation, (2) spatially encoding dosimetric goals to convey clinical intent, and (3) incorporating plan‐sum data as model inputs. Collectively, this approach enables the training of a universal, fraction‐agnostic and site‐agnostic deep learning model with course‐wide context for dose prediction. Importantly, our framework extends naturally to single‐plan courses by simply substituting plan‐specific goals and leaving the plan‐sum channel empty.

Several challenges were encountered during model development and training. Firstly, although our single‐plan baseline model deliberately omitted most plan‐sum context, it still benefitted from inherent bias in the dataset. All clinical plans were designed with cumulative plan‐sum constraints in mind, and all goals used for input were re‐scaled from plan‐sum objectives rather than derived independently. Consequently, the single‐plan model's performance may be inflated relative to a fully plan‐independent baseline trained without plan‐sum‐derived goals. The gap reported in Tables 2, 3, 4 is therefore likely to be a lower bound on the benefit of plan‐sum context. We believe this represents an appropriate and conservative baseline comparison: it isolates the contribution of the multi‐plan architecture, so any advantage conferred to the single‐plan model by this implicit plan‐sum information makes the multi‐plan model's superior performance more meaningful, not less.

Secondly, our patient‐centric batching strategy was intentionally designed for course‐centric parameter updates. In this design, the single‐plan model was blinded to only a single plan per batch, so it used three augmented samples from a single plan per epoch compared to the multi‐plan model's three augmented samples from at least two distinct plans. This approach may have impacted the single‐plan model's generalizability. Thirdly, the multi‐task nature of the multi‐plan model led to gradient imbalances during training, often dominated by the plan‐sum task due to larger dose values. This imbalance may have skewed multi‐plan model optimization towards plan‐sum predictions, but this can be addressed in the future via task‐specific loss weighting or hyperparameter tuning.

Despite utilizing a dataset of modest size (131 plans across 64 patients), its diversity allowed us to evaluate sparsely represented disease sites (see Figures 9, 10). The site imbalance in our dataset (37 H&N versus 1–2 cases for some sites) raises a natural question about whether performance is driven primarily by well‐represented sites. However, our results suggest that the lung and prostate cases in the test set (Figures 9, 10) achieved clinically acceptable predictions despite minimal representation in training. As described previously, the goal array encodes planning objectives in place of fixed OAR‐specific input channels, which may enable knowledge learned from data‐rich sites to inform predictions for data‐sparse sites. While we cannot definitively establish the minimum training cases required per site from this study alone, our results suggest that even single‐case representation may be sufficient when combined with adequate representation from other sites. Formal characterization of the relationship between per‐site sample size and prediction accuracy remains an area for future investigation with larger, more balanced datasets. Additionally, explicit validation of cross‐site generalization by holding out entire treatment site(s) during training would provide stronger evidence for the model's ability to predict dose for anatomical regions not seen during training.

In regards to our preprocessing strategy for generating spatially encoded dosimetric goals, we defaulted to readily available institutional guidelines based on disease site and fractionation scheme and superseded them with a physician's patient‐specific directive when one was available. For broader application when such resources are unavailable, published consensus guidelines (e.g., the QUANTEC effort) could serve as a source for organ‐sparing goals.^22^ It is crucial to clarify that these goals represent intended constraints, not the final achieved dose. Inclusion of these goals is essential to universal dose prediction, as it explicitly informs the model of dosimetric intent rather than relying solely on implicit assumptions. Additionally, data augmentation strategies were specially designed to prevent overfitting to specific goals or OAR values, and to enhance model robustness and generalization.

One limitation of this approach is that certain site‐specific planning practices are not captured in formal planning objectives but nonetheless influence clinical dose distributions. For example, some site‐specific planning practices prioritize minimization of dose to specific OARs far beyond their objectives (e.g., sparing rectum volume from 30% prescription dose). Additional failure modes in which dose predictions may be suboptimal include: re‐irradiation cases in which prior delivered dose is not represented in the dosimetric goals, and cases with prescription levels outside the trained range that have not yet been validated. Future work could explore encoding these practices in the goal array to better reflect site‐specific planning conventions, and explicitly validate the model on re‐irradiation and outlier‐prescription cases.

In summary, this study demonstrates the tangible benefits of incorporating cumulative plan‐sum information into a multi‐task dose prediction framework for sequential boost RT. Through simultaneous prediction of individual plan and plan‐sum doses, our multi‐plan model offers a pathway to treatment planning by providing physicians and planners with an accurate and comprehensive strategy for dose allocation upfront. Unlike conventional single‐plan models, which cannot account for inter‐plan interactions in sequential treatments, our approach provides course‐wide context that enables more informed optimization. This pre‐planning guidance may reduce the number of iterations required for planners to achieve clinically acceptable treatment plans, enhance treatment planning consistency, and improve overall plan quality by minimizing reliance on trial‐and‐error inference during optimization.

Advancing this multi‐objective, multi‐plan framework towards a truly universal dose prediction model offers significant advantages. Primarily, it eliminates the complexity and resource burden associated with developing and maintaining numerous specialized models. Furthermore, this universality enables training on substantially larger datasets, potentially enhancing model robustness and generalizability—even in traditionally data‐limited scenarios. Compared to typical specialized dose prediction models, which generally use CT images, OARs, and PTVs as inputs, our framework only requires the addition of organ‐sparing goals—which is an input that can be standardized or automated using templates.

## 5 CONCLUSIONS

We developed a multi‐objective, multi‐plan deep learning framework for dose prediction in sequential boost RT that jointly predicts individual plan and cumulative plan‐sum dose distributions. The model incorporates spatially encoded dosimetric goals, a consolidated OAR representation, and cumulative plan‐sum inputs, enabling a context‐aware strategy that accounts for inter‐plan trade‐offs and cumulative dose requirements otherwise inaccessible to conventional single‐plan frameworks. Compared to a single‐plan model, our multi‐plan approach demonstrated improvements across voxel‐level, DVH‐based, and structural similarity metrics for both plan and plan‐sum dose distributions.

The multi‐plan model can provide accurate, comprehensive pre‐planning guidance to planners and physicians, with potential to reduce the number of optimization iterations required, enhance plan consistency, and improve overall plan quality. More broadly, this work outlines a strategy for training a universal dose prediction model with the potential to generalize across disease sites, fractionation schemes, and any number of plans within a treatment course. Future directions include expanding this work to larger and more diverse datasets to robustly validate universal generalization capabilities, as well as subsequent integration of this framework into our clinical workflow.

## CONFLICT OF INTEREST STATEMENT

The authors declare no conflicts of interest.

## Figures and Tables

**FIGURE A1:**
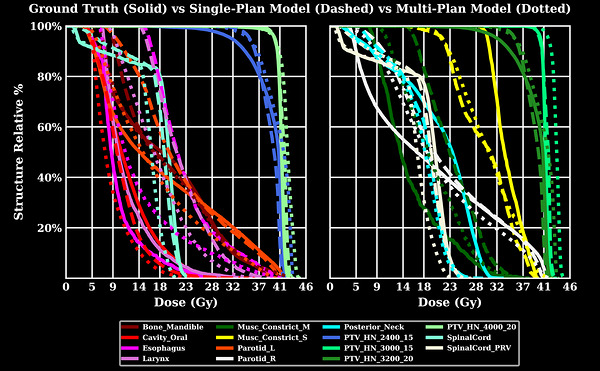
DVH comparison corresponding to Figure 6 (H&N initial plan, 40 Gy in 20 fractions). Ground truth: solid lines. Single‐plan model: dashed lines. Multi‐plan model: dotted lines.

**FIGURE A2:**
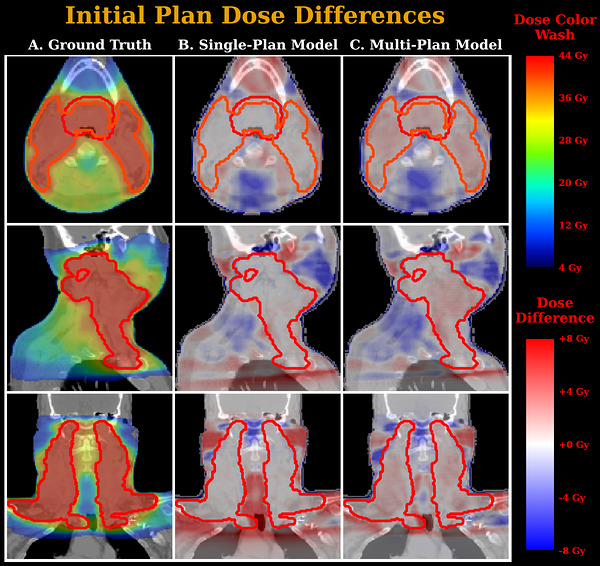
Dose difference comparison corresponding to Figure 6 (H&N initial plan, 40 Gy in 20 fractions). Panels (a)–(c) represent the clinically approved ground truth, the single‐plan model prediction difference from ground truth, and the multi‐plan model prediction difference from ground truth, respectively. Differences are displayed from −20% to +20% of prescription dose, with red indicating overprediction and blue indicating overprediction.

**FIGURE A3:**
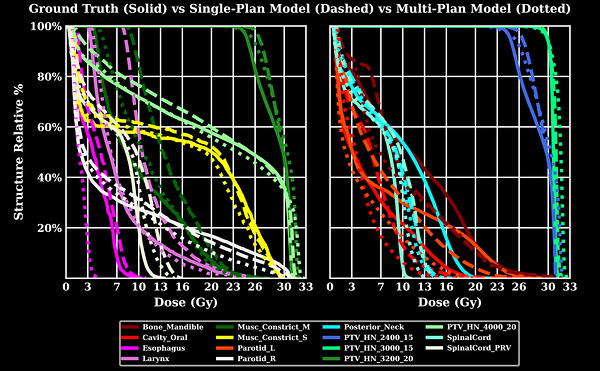
DVH comparison corresponding to Figure 7 (H&N boost plan, 30 Gy in 15 fractions). Ground truth: solid lines. Single‐plan model: dashed lines. Multi‐plan model: dotted lines.

**FIGURE A4:**
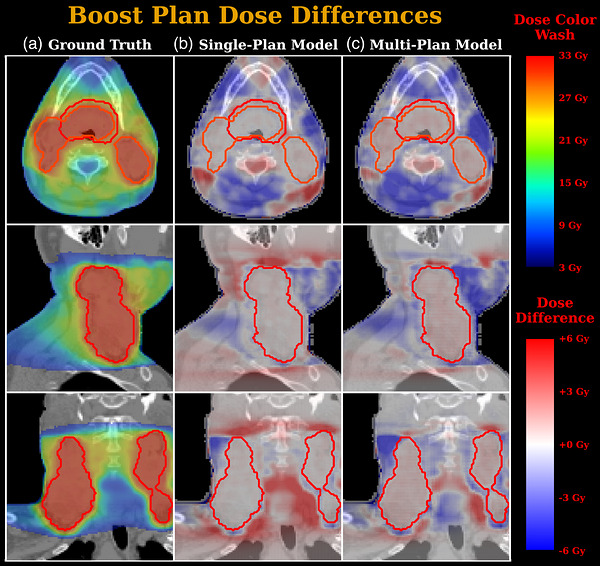
Dose difference comparison corresponding to Figure 7 (H&N boost plan, 30 Gy in 15 fractions). Panels (a)–(c) represent the clinically approved ground truth dose, the single‐plan model prediction difference from ground truth, and the multi‐plan model prediction difference from ground truth, respectively. Differences are displayed from −20% to +20% of prescription dose, with red indicating overprediction and blue indicating underprediction.

**FIGURE A5:**
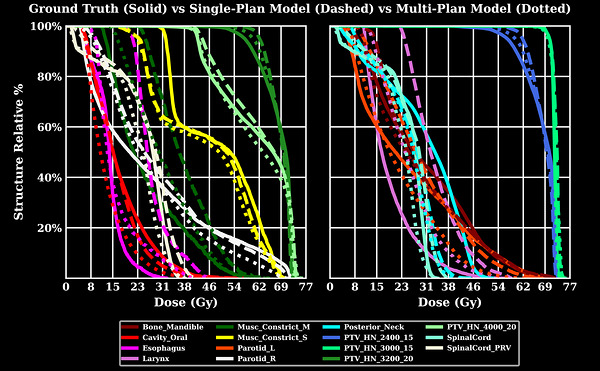
DVH comparison corresponding to Figure 8 (H&N plan sum, total of 70 Gy in 35 fractions). Ground truth: solid lines. Single‐plan model: dashed lines. Multi‐plan model: dotted lines.

**FIGURE A6:**
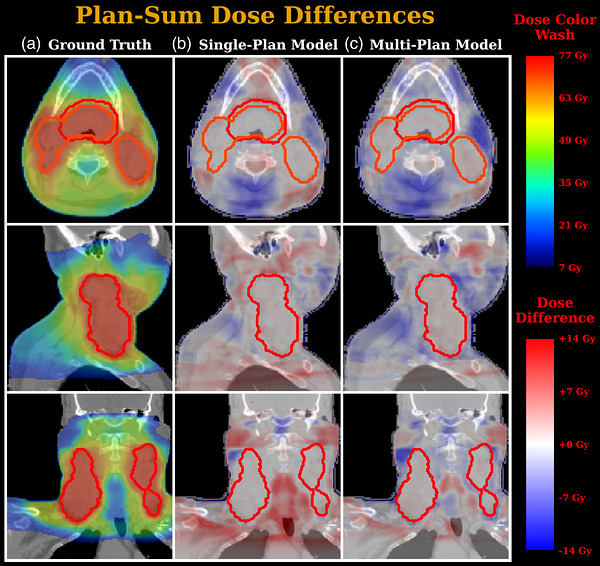
Dose difference comparison corresponding to Figure 8 (H&N plan sum, total of 70 Gy in 35 fractions). Panels (a)–(c) represent the clinically approved ground truth dose, the single‐plan model prediction difference from ground truth, and the multi‐plan model prediction difference from ground truth, respectively. Differences are displayed from −20% to +20% of prescription dose, with red indicating overprediction and blue indicating underprediction.

**FIGURE A7:**
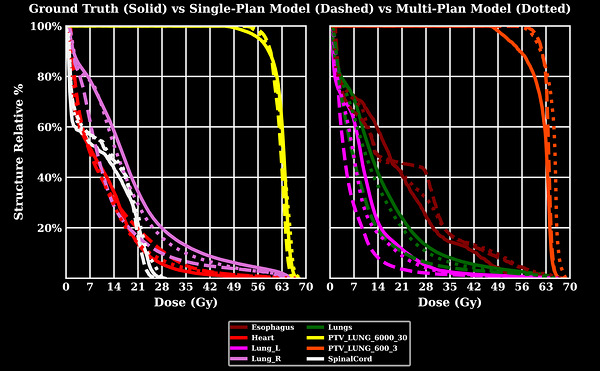
DVH comparison corresponding to Figure 9 (Lung initial plan, 60 Gy in 30 fractions). Ground truth: solid lines. Single‐plan model: dashed lines. Multi‐plan model: dotted lines.

**FIGURE A8:**
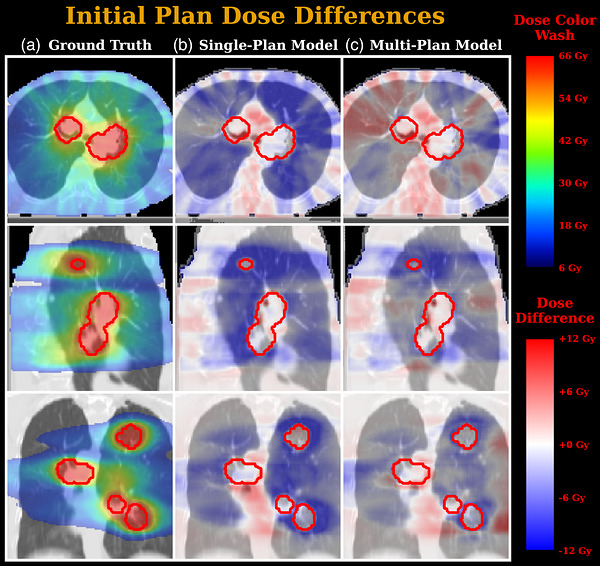
Dose difference comparison corresponding to Figure 9 (Lung initial plan, 60 Gy in 30 fractions). Panels (a)–(c) represent the clinically approved ground truth dose, the single‐plan model prediction difference from ground truth, and the multi‐plan model prediction difference from ground truth, respectively. Differences are displayed from −20% to +20% of prescription dose, with red indicating overprediction and blue indicating underprediction.

**FIGURE A9:**
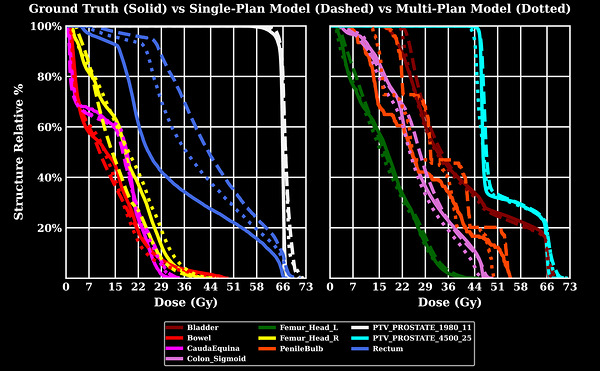
DVH comparison corresponding to Figure 10 (Prostate plan sum, total of 64.8 Gy in 36 fractions). Ground truth: solid lines. Single‐plan model: dashed lines. Multi‐plan model: dotted lines.

**FIGURE A10:**
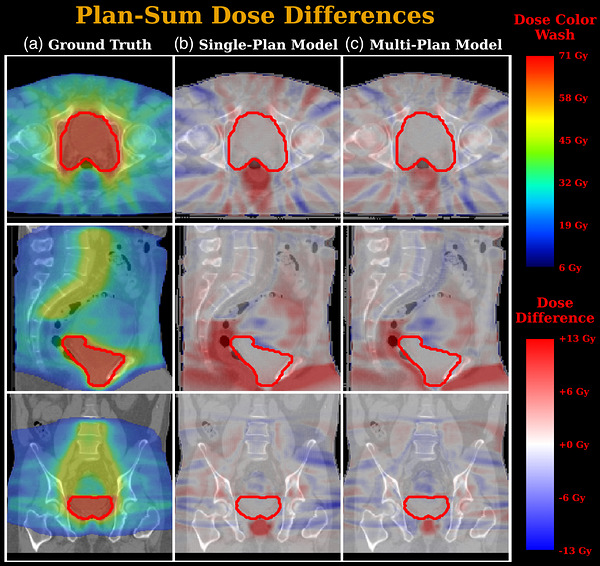
Dose difference comparison corresponding to Figure 10 (Prostate plan sum, total of 64.8 Gy in 36 fractions). Panels (a)–(c) represent the clinically approved ground truth dose, the single‐plan model prediction difference from ground truth, and the multi‐plan model prediction difference from ground truth, respectively. Differences are displayed from −20% to +20% of prescription dose, with red indicating overprediction and blue indicating underprediction.

## Data Availability

Authors will share data upon request to the corresponding author.
